# A molecular approach to the genus *Alburnoides* using COI sequences data set and the description of a new species, *A. damghani*, from the Damghan River system (the Dasht-e Kavir Basin, Iran) (Actinopterygii, Cyprinidae)

**DOI:** 10.3897/zookeys.579.7665

**Published:** 2016-04-11

**Authors:** Arash Jouladeh Roudbar, Soheil Eagderi, Hamid Reza Esmaeili, Brian W. Coad, Nina Bogutskaya

**Affiliations:** 1 Department of Fisheries, Faculty of Natural Resources, Sari University of Agricultural Sciences and Natural Resources, Sari, Iran; 2 Department of Fisheries, Faculty of Natural Resources, University of Tehran, Karaj, Iran; 3 Ichthyology Research Lab., Department of Biology, College of Sciences, Shiraz University, Shiraz, 71454–Iran; 4 Canadian Museum of Nature, Ottawa, Ontario, Canada, K1P 6P4; 5 Natural History Museum, Vienna, Austria

**Keywords:** Freshwater fishes, phylogenetic relationship, mitochondrial DNA, DNA barcoding, morphology

## Abstract

The molecular status of nine species of the genus *Alburnoides* from different river drainages in Iran and additionally by seven species from Europe was assessed. mtDNA COI gene sequences from freshly collected specimens and available NCBI data revealed four major phylogenetic lineages. Based on the results, a distinct taxon from the Cheshmeh Ali (Ali Spring), a Damghan River tributary in the endorheic Dasht-e Kavir basin, northern Iran, which is the closest sister to *Alburnoides namaki* (Namak Lake basin) + *Alburnoides coadi* (Nam River in the endorheic Dasht-e Kavir basin) is considered as a new species, *Alburnoides damghani* **sp. n.** It is distinguished from other *Alburnoides* species in Iran by a combination of character states including: a weakly-developed, variably-scaled, ventral keel from completely scaleless to completely scaled, a short snout with the tip of the mouth cleft on a level with the lower margin of the pupil or slightly lower, a small eye (eye horizontal diameter slightly to markedly less than interorbital width), commonly 8½ branched dorsal-fin rays, commonly 11−12½ branched anal-fin rays, 40−46(47) total lateral-line scales, 2.5–4.2 or 2.5–4.1 pharyngeal teeth, gill rakers short and widely spaced, 6−8 in total, 39−41 (commonly 40), total vertebrae, (19)20(21) abdominal vertebrae, 19−21 (most commonly 20) caudal vertebrae, abdominal vertebral region most commonly equal to or longer than caudal region, and most common vertebral formulae 20+20 and 21+19.

## Introduction

The genus *Alburnoides*, a member of the family Cyprinidae, is found in Europe, Asia Minor and Central Asia with 28 species so far considered valid (Bogutskaya and Coad 2009, Coad and Bogutskaya 2009, 2012, Turan et al. 2014, Mousavi-Sabet et al. 2015a, b, Coad 2015). *Alburnoides bipunctatus* (Bloch, 1782) was the name applied to most populations throughout Europe and the Middle East from north of the Alps (France) eastwards to the Black, Caspian and Aral Sea basins but ongoing research has revealed a much greater diversity (Bogutskaya and Coad 2009, Coad and Bogutskaya 2009, Seifali et al. 2012, Turan et al. 2014, Mousavi-Sabet et al. 2015a, b).

**Figure 1.**
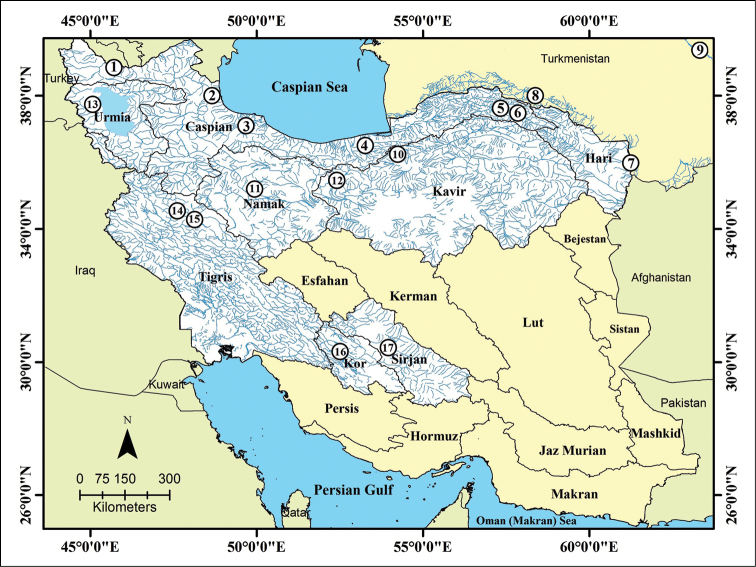
Distribution and sampling sites of *Alburnoides* species in Iran and adjacent areas. **1** *Alburnoides eichwaldii*: Aras River, Kura River drainage **2** Alburnoides cf. eichwaldii: west of Safid River **3** *Alburnoides samiii*: Safid River **4** *Alburnoides tabarestanensis*: Tajan River **5** *Alburnoides parhami*: Atrek River **6** *Alburnoides parhami*: type locality Baba-Aman stream **7** *Alburnoides holciki*: Hari River **8** *Alburnoides varentsovi*: Ashkhabadka River, northern slope of Kopetdag Mountains **9** *Alburnoides* sp. Amu Darya River **10** *Alburnoides damghani* sp. n.: Cheshmeh Ali, Damghan River system, Dasht-e Kavir basin **11** *Alburnoides namaki*: Qarah River, Namak Lake basin **12** *Alburnoides coadi*: Nam River, Dasht-e Kavir basin **13** *Alburnoides petrubanarescui*: Orumiyeh Lake basin **14** *Alburnoides nicolausi*: Nor Abad River, Tigris River system **15** *Alburnoides idignensis*: Bid Sorkh River, Tigris River system **16** *Alburnoides qanati*: Pulvar River, Kor River system **17** *Alburnoides qanati*: Masih Spring, Sirjan basin.

Based on recent research, eleven species were considered to occur in Iranian inland waters. First, *Alburnoides eichwaldii* (De Filippi, 1863) from the Kura River drainage was resurrected (Bogutskaya and Coad 2009) and six species described: *Alburnoides namaki* Bogutskaya & Coad, 2009 from a qanat at Taveh, Namak Lake basin, *Alburnoides nicolausi* Bogutskaya & Coad, 2009 from the Tigris River drainage, *Alburnoides qanati* Coad & Bogutskaya, 2009 from the Pulvar River drainage, Kor River basin, *Alburnoides idignensis* Bogutskaya & Coad, 2009 from the Bid Sorkh River, Gav Masiab River system, Tigris River drainage, *Alburnoides petrubanarescui* Coad & Bogutskaya, 2009 from the Qasemlou Chay, Orumiyeh (Urmia) Lake basin, and *Alburnoides holciki* Coad & Bogutskaya, 2012 from the Hari River. It was also shown (Coad and Bogutskaya 2009, 2012) that south-Caspian *Alburnoides* from 1) rivers west of the Safid River [Sefid Rud]; 2) the Safid River drainage; 3) rivers east of the Safid River excluding the Atrek [Atrak] drainage; 4) the Atrek River drainage; and 5) the Amu Darya River drainage represent undescribed species. It was expected that even more species are to be recognized (see Coad and Bogutskaya 2012, Seifali et al. 2012). *Alburnoides* sp. from the Tajan River (*Alburnoides* sp. from rivers east of the Safid River *sensu* Coad and Bogutskaya (2009)) was later described as *Alburnoides tabarestanensis* Mousavi-Sabet, Anvarifar & Azizi, 2015; *Alburnoides* sp. from the Safid River was described as *Alburnoides samiii* Mousavi-Sabet, Vatandoust & Doadrio, 2015, and the Atrek River *Alburnoides* sp. as *Alburnoides parhami* Mousavi-Sabet, Vatandoust & Doadrio, 2015. Distribution of the species is given in Fig. 1.

A comparison of populations of different *Alburnoides* species and unidentified populations based on molecular characteristics, body, head and mouth shape, the ventral keel development, and meristic characters showed that a population from Cheshmeh Ali, a Damghan River tributary in the Dasht-e Kavir drainage, could not be identified with any of the named species and represents a new species. Hence, the aim of this study was to describe this new species based on a wide comparison with known Iranian species of the genus and investigate phylogenetic relationships among the major *Alburnoides* lineages by analyzing sequence variation of the mitochondrial COI gene.

## Materials and methods

### Morphological examinations

After anesthesia, fishes were fixed in 5% formaldehyde and later stored in 70% ethanol. Counts and measurements follow Hubbs and Lagler (1958). Measurements were performed using digital calipers to the nearest 0.01 mm. Standard length (SL) was measured from the tip of the upper jaw to the end of the hypural complex, total length (TL) was measured from the tip of the upper jaw to the end of the longest caudal-fin lobe. Head length and interorbital width were measured to their bony margins. Fin ray counts separate unbranched and branched rays. The last two branched rays articulated on a last compound pterygiophore in the dorsal and anal fins and are noted as “1½”. Mean and standard deviation were calculated without the “½”. Lateral-line scale count includes pierced scales, from the first one just behind the supracleithrum to the posteriormost one at the base of the caudal-fin rays (i.e., posterior margin of the hypurals) excluding 1 or 2 scales located on the bases of the caudal-fin rays, total number of lateral-line scales is also provided. Counts of unpaired fin rays and vertebrae were done from radiographs. The character states of the ventral keel scale cover were estimated by direct measurements as shown in Bogutskaya et al. (2010). Statistical calculations and the multidimensional scaling (MDS) analysis were performed using software packages Statistica 6.0 and Primer v6.1.9.

### DNA extraction and PCR

DNA was extracted from muscle tissue at the base of the dorsal fin using a Genomic DNA Purification Kit (#K0512, Thermo Scientific Corporation, Lithuania) following the manufacturer’s protocol. The COI gene was amplified using primers FishF1-(5'-TCAACCAACCACAAAGACATTGGCAC-3') and FishR1-(5'-TAGACTTCTGGGTGGCCAAAGAATCA-3'), designed by Ward et al. (2005). Polymerase chain reaction (PCR) conditions were as follows: a 50 μl final reaction volume containing 5 μl of 10X Taq polymerase buffer, 1 μl of (50 mM) MgCl_2_,1 μl of (10 mM) deoxynucleotide triphosphate (dNTP), 1 μl (10 μm) of each primer, 1 μl of Taq polymerase (5 Uμl^-1^), 7 μl of total DNA and 33 μl of H_2_O. Amplification cycles were as follows: denaturation for 10 min at 94 °C, 30 cycles at 94 °C for 1 min, 58.5 °C for 1 min, 72 °C for 1 min and a final extension for 5 min at 72 °C. PCR products were purified using a purification kit (Expin Combo GP – mini, Macrogen Inc., Korea). The PCR products were sequenced using the Sanger method by a robotic ABI-3130xl sequencer using manufacturer’s protocol. The forward primer FishF1 was used for single strand sequencing.

### Molecular data analysis

The haplotypes were compared to published *Alburnoides* sequences using (BLASTn) basic local alignment search tool (Altschul et al. 1990). All sequence data were aligned using MEGA6 software (Tamura et al. 2013). To unify the length of the sequences, the common 620 bp length segments were selected and used for phylogenetic analysis. Modeltest (Posada and Crandall 1998), implemented in the MEGA 6 software (Tamura et al. 2013), was used to determine the most appropriate sequence evolution model for the given data, treating gaps and missing data with the partial deletion option under 95% site coverage cut-off. We generated maximum likelihood phylogenetic trees with 10,000 bootstrap replicates in RaxML software 7.2.5 Stamatakis (2006) under the GTR+G+I model of nucleotide substitution, with CAT approximation of rate heterogeneity and fast bootstrap to explore species phylogenetic affinities. Bayesian analyses of nucleotide sequences were run with the parallel version of MrBayes 3.1.2 (Ronquist and Huelsenbeck 2003) on a Linux cluster with one processor assigned to each Markov chain under the most generalizing model (GTR+G+I) because overparametrization apparently does not negatively affect Bayesian analyses (Huelsenbeck and Ranala 2004). Each Bayesian analysis comprised two simultaneous runs of four Metropolis-coupled Markov-chains at the default temperature (0.2). Analyses were terminated after the chains converged significantly, as indicated by the average standard deviation of split frequencies <0.01.

Sequenced were Iranian populations of *Alburnoides coadi*, *Alburnoides damghani* sp. n., *Alburnoides eichwaldii*, *Alburnoides holciki*, *Alburnoides idignensis*, *Alburnoides namaki*, *Alburnoides nicolausi*, *Alburnoides qanati*, *Alburnoides samiii* and *Alburnoides tabarestanensis* (Fig. 1, Table 1). No tissue material was available for *Alburnoides petrubanarescui*. In order to better understand the phylogenetic position of the studied species, we included records from the NCBI GenBank for *Alburnoides bipunctatus* (accession numbers: KJ552394, KM286434, KM286435, KJ552440, 286433), *Alburnoides devolli* Bogutskaya, Zupančič & Naseka 2010 (accession numbers: KJ552420, KJ552652, KJ552693, KJ552370), *Alburnoides fangfangae* Bogutskaya, Zupančič & Naseka, 2010 (accession numbers: KJ552562, KJ552720, *Alburnoides* KJ552616, KJ552506,) *Alburnoides ohridanus* Karaman, 1928 (accession numbers: KJ552755, KJ552448, KJ552646, KJ552730), *Alburnoides prespensis* Karaman, 1924 (accession numbers: KJ552408, HQ600666A, HQ600665, KJ552526, KJ552408, *Alburnoides* HQ600667), *Alburnoides strymonicus* Chichkoff, 1940 (accession numbers: KJ552519, KJ552521), *Alburnoides thessalicus* Stephanidis, 1950 (accession numbers: KJ552656, KJ552369, KJ552723, KJ552685) and *Alburnoides* sp. (accession number: KJ552427, Greece: Sperchios drainage).

**Table 1.** Details of the specimens used for molecular analysis.

Species	Accession No.	Sampling site	Latitude	Longitude	Basin/drainage
*Alburnoides damghani 1*	KU705237	Damghan Spring	36°16'45.6"	54°05'01.6"	Dasht-e Kavir
*Alburnoides damghani 2*	KU705238	Damghan Spring	36°16'45.6"	54°05'01.6"	Dasht-e Kavir
*Alburnoides damghani 3*	KU705239	Damghan Spring	36°16'45.6"	54°05'01.6"	Dasht-e Kavir
*Alburnoides eichwaldii* 7	KU705240	Aras River	39°21'07"	45°05'08"	Caspian Sea
*Alburnoides eichwaldii* 8	KU705241	Aras River	39°21'07"	45°05'08"	Caspian Sea
*Alburnoides eichwaldii* 9	KU705242	Aras River	39°21'07"	45°05'08"	Caspian Sea
*Alburnoides eichwaldii* 38	KU705243	Aras River	39°35'02"	47°42'35"	Caspian Sea
*Alburnoides holciki* 22	KU705244	Hari River	35°05'	61°08'	Hari River
*Alburnoides holciki* 23	KU705245	Hari River	35°05'	61°08'	Hari River
*Alburnoides holciki* 24	KU705246	Hari River	35°05'	61°08'	Hari River
*Alburnoides idignensis* 4	KU705247	Bid Sorkh River	34°23'	47°52'	Tigris River
*Alburnoides idignensis* 5	KU705248	Bid Sorkh River	34°23'	47°52'	Tigris River
*Alburnoides idignensis* 6	KU705249	Bid Sorkh River	34°23'	47°52'	Tigris River
*Alburnoides idignensis* 34	KU705250	Chardavol River	33°41'38"	46°52'57"	Tigris River
*Alburnoides namaki* 16	KU705251	Qareh Chai River	34°53'	50°02'	Namak Lake
*Alburnoides namaki* 17	KU705252	Qareh Chai River	34°53'	50°02'	Namak Lake
*Alburnoides namaki 18*	KU705253	Qareh Chai River	34°53'	50°02'	Namak Lake
*Alburnoides namaki* 31	KU705254	Doab River	34°04'20"	49°20'46"	Namak Lake
*Alburnoides namaki* 32	KU705255	Doab River	34°04'20"	49°20'46"	Namak Lake
*Alburnoides coadi* 1	KU705256	Nam River	35°43'21"	52°39'20"	Dasht-e Kavir
*Alburnoides coadi* 2	KU705257	Nam River	35°43'21"	52°39'20"	Dasht-e Kavir
*Alburnoides coadi* 3	KU705258	Nam River	35°43'21"	52°39'20"	Dasht-e Kavir
*Alburnoides nicolausi* 10	KU705259	Nor Abad River	34°03'	47°58'	Tigris River
*Alburnoides nicolausi* 11	KU705260	Nor Abad River	34°03'	47°58'	Tigris River
*Alburnoides nicolausi* 12	KU705261	Nor Abad River	34°03'	47°58'	Tigris River
*Alburnoides qanati* 13	KU705262	Pulvar River	29°59'	52°54'	Kor River
*Alburnoides qanati* 14	KU705263	Pulvar River	29°59'	52°54'	Kor River
*Alburnoides qanati* 15	KU705264	Pulvar River	29°59'	52°54'	Kor River
*Alburnoides qanati* 39	KU705265	Ghadamgah Spring	30°14'20"	52°22'23"	Kor River
*Alburnoides qanati* 40	KU705266	Herat (Masih Spring)	30°01'57"	54°19'55"	Sirjan
*Alburnoides tabarestanensis* 19	KU705267	Tajan River	36°11'	53°19'	Caspian Sea
*Alburnoides tabarestanensis* 20	KU705268	Tajan River	36°11'	53°19'	Caspian Sea
*Alburnoides tabarestanensis* 21	KU705269	Tajan River	36°11'	53°19'	Caspian Sea
*Alburnoides tabarestanensis* 25	KU705270	Tajan River	36°16'37"	53°12'22"	Caspian Sea
*Alburnoides samiii* 26	KU705271	Emamzadeh Hashem (Safid River)	37°01'11"	49°38'	Caspian Sea
*Alburnoides samiii* 27	KU705272	Chalavand River	38°17'39"	48°52'28"	Caspian Sea

Screening for diagnostic nucleotide substitutions relative to *Oryzias latipes* was performed manually from the resulting sequence alignment. Estimates of evolutionary divergence over sequence pairs between species were conducted in Mega6 (Tamura et al. 2013). Analyses were conducted using the Kimura 2-parameter model (Kimura 1980). The rate variation among sites was modelled with a gamma distribution (shape parameter = 1). Codon positions included were 1st+2nd+3rd. All positions containing gaps and missing data were eliminated.

As appropriate outgroup to root the constructed phylogenetic hypothesis, *Alburnus alburnus* (accession number: KM373683), was included.

### Abbreviations used

SL, standard length, HL, lateral head length, K2P, Kimura 2-parameter.

*Collection codes*: CMNFI – Canadian Museum of Nature, Ottawa, ZM-CBSU – Zoological Museum of Shiraz University, Collection of Biology Department, Shiraz.

## Results

**Figure 2.**
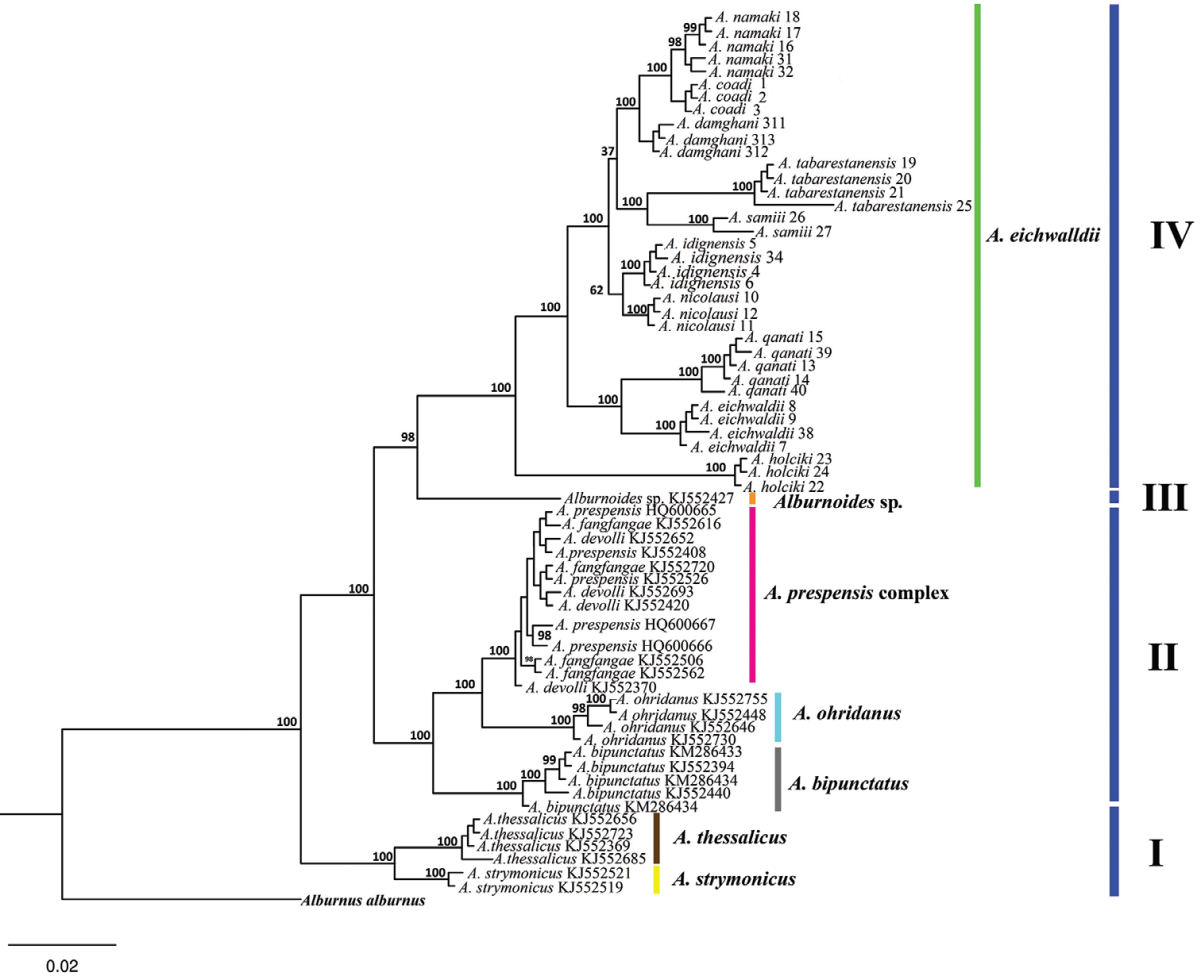
Bayesian analysis (based on *COI* gene sequences) of phylogenetic relationships of *Alburnoides damghani* and related taxa.

COI barcodes were generated for a total of 36 *Alburnoides* specimens. Two phylogenetic approaches Bayesian Inference (BI) and Maximum Likelihood (ML), gave the same tree topologies and thus one is presented (Fig. 2). Tables 2–3 list the diagnostic nucleotide substitutions and estimates of the average evolutionary divergence found in the mtDNA COI barcode region. The two different phylogenetic approaches produced almost identical tree topologies although Bayesian analysis (Rannala and Yang 1986, Yang and Rannala 1997) has been empirically demonstrated to be the most efficient character-based method for accurately reconstructing a phylogeny (Simmons and Miya 2004). Two methods produced trees with 4 major lineages supported by high posterior probability and bootstrap values and seven groups (Fig. 2): I) *Alburnoides strymonicus − Alburnoides thessalicus* lineage, II) *Alburnoides bipunctatus* − *Alburnoides ohridanus* − *Alburnoides prespensis* group lineage, III) *Alburnoides* sp. lineage (Greece: Sperchios drainage) and IV) Iranian *Alburnoides* lineage (*Alburnoides eichwaldii* lineage). Within the IV line, *Alburnoides damghani* sp. n. is a sister to *Alburnoides namaki* + *Alburnoides coadi* and the clade containing the three species is a sister to *Alburnoides tabarestanensis* + *Alburnoides samiii* (Fig. 2).

**Table 2.** Diagnostic nucleotide substitutions found in the mtDNA COI barcode region of *Alburnoides* species of Iran.

	Nucleotide position relative to *Oryzias latipes* complete mitochondrial genome (AP004421)																							
	N	5519	5529	5532	5535	5538	5598	5601	5631	5649	5667	5673	5679	5682	5691	5694	5700	5701	5707	5713	5722	5731	5734	5765
*Alburnoides eichwaldii*	4	T	G	C	A	A	A	**C**	T	A	G	T	T	**T**	A	C	C	G	**G**	G	A	A	A	C
*Alburnoides damghani*	3	T	G	C	A	A	A	A	T	A	G	T	T	A	A	C	C	G	A	G	A	A	A	C
*Alburnoides holciki*	3	**A**	G	**T**	A	A	A	A	T	A	G	T	T	A	A	C	C	**A**	A	G	**C**	**G**	A	**T**
*Alburnoides idignensis*	4	T	G	C	A	A	A	A	T	**G**	G	T	T	A	A	C	C	G	A	G	A	A	A	C
*Alburnoides namaki*	8	T	G	C	A	**G**	A	A	T	A	G	T	T	A	A	C	C	G	A	G	A	A	A	C
*Alburnoides nicolausi*	3	T	G	C	A	A	A	A	T	A	G	T	T	A	A	C	C	G	A	G	A	A	A	C
*Alburnoides qanati*	5	T	**A**	C	A	A	A	A	**C**	A	G	T	T	**C**	A	**T**	C	G	A	**A**	A	A	**G**	C
*Alburnoides samiii*	2	T	G	C	A	A	A	A	T	A	G	T	T	A	**G**	C	C	G	A	G	**G**	A	A	C
*Alburnoides tabarestanensis*	4	T	G	C	**G**	A	**G**	A	T	A	**A**	**C**	**C**	**G**	A	C	**T**	G	A	G	A	A	A	C
	N	5776	5786	5789	5800	5815	5818	5821	5836	5854	5857	5885	5902	5911	5914	5920	5959	5965	5992	6001	6004	6019	6050	6092
*Alburnoides eichwaldii*	4	G	C	C	T	T	A	C	A	**G**	A	G	A	A	A	**G**	T	T	T	C	C	G	T	C
*Alburnoides damghani*	3	G	C	C	T	T	G	C	A	A	A	G	A	A	A	A	T	T	T	T	C	G	T	C
*Alburnoides holciki*	3	G	**T**	**T**	T	T	**C**	**T**	A	A	**T**	G	A	**G**	**G**	A	T	**C**	**C**	**G**	**A**	**A**	**C**	C
*Alburnoides idignensis*	4	G	C	C	T	T	A	C	A	A	A	G	A	A	A	A	T	T	T	C	C	G	T	C
*Alburnoides namaki*	8	G	C	C	T	T	A	C	A	A	A	G	A	A	A	A	**C**	T	T	T	C	G	T	C
*Alburnoides nicolausi*	3	G	C	C	T	T	A	C	A	A	A	**A**	**G**	A	A	A	T	T	T	T	C	G	T	C
*Alburnoides qanati*	5	G	C	C	T	T	G	C	**G**	A	A	G	A	A	A	A	T	T	T	C	C	G	T	**T**
*Alburnoides samiii*	2	G	C	C	T	**C**	A	C	A	A	A	G	A	A	A	A	T	T	T	T	C	G	T	C
*Alburnoides tabarestanensis*	4	**A**	C	C	**C**	T	A	C	A	A	A	G	A	A	A	A	T	T	T	T	C	G	T	C

**Table 3.** Estimates of the average evolutionary divergence between Iranian *Alburnoides* species, expressed as number of base substitutions per site. All positions with less than 95% site coverage were eliminated before analysis, leading to a total of 620 nucleotide positions.

No.	Species	N	1	2	3	4	5	6	7	8
1	*Alburnoides eichwaldii*	4								
2	*Alburnoides damghani*	3	3.08							
3	*Alburnoides holciki*	3	6.78	5.75						
4	*Alburnoides idignensis*	4	2.76	1.08	5.70					
5	*Alburnoides namaki*	8	3.74	0.97	6.18	1.72				
6	*Alburnoides nicolausi*	3	3.08	1.04	5.94	0.73	1.68			
7	*Alburnoides qanati*	5	2.90	3.57	7.12	3.62	4.61	3.94		
8	*Alburnoides samiii*	2	4.21	2.54	6.41	2.58	3.19	2.54	5.52	
9	*Alburnoides tabarestanensis*	4	5.09	3.17	7.76	3.21	3.83	3.17	5.99	3.64

### Alburnoides damghani sp. n.

Taxon classificationAnimaliaCypriniformesCyprinidae

http://zoobank.org/BD1CFF35-5F9F-4823-ABC9-E4A9FA5D990E

Figs 3 , 4 , 5 , 6

#### Type locality.

Cheshmeh Ali (Ali Spring), Damghan River tributary, Iran.

#### Holotype.

CMNFI 2015-0091, female, 67.0 mm SL, Iran, Semnan Prov., Cheshmeh Ali, Damghan River tributary, near Damghan city, Dasht-e Kavir Basin, 36°16'45.6"N, 54°05'01.6"E, altitude 1569 m, 22 August 2011, coll. H.R. Esmaeili, A. Gholamifard, G. Sayyadzadeh, R. Zamaniannejad.

#### Paratypes.

ZM-CBSU 2011-1, 15 specimens, 57.1−79 mm SL, same data as holotype; CMNFI 2015-0091A, 24 specimens, 54.6−84.4 mm SL, same data as holotype; ZM-CBSU 2012-1, 3 specimens, 83.9−89.7 mm SL, same data as holotype, 06 July 2012, coll. S. Eagderi.

#### Diagnosis.

*Alburnoides damghani* sp. n. is distinguished by having a combination of character states which includes a weakly-developed, variably-scaled, ventral keel from completely scaleless to completely scaled; a stout short snout with tip of the mouth cleft on a level with the lower margin of the pupil or lower; a small eye (eye horizontal diameter slightly to markedly less than interorbital width); commonly 8½ branched dorsal-fin rays; commonly 11−12½, branched anal-fin rays; 40−46(47) total lateral-line scales (40-46 scales to posterior margin of the hypurals); 2.5–4.2 and 2.5-4.1 pharyngeal teeth; 6−8 total gill rakers in outer row on first left arch; 39−41, commonly 40, total vertebrae; 12−14, commonly 13, predorsal vertebrae; abdominal vertebral region most commonly equal to or longer than caudal region (vertebral formulae 20+20 and 21+19).

#### Description.

**Figure 3.**
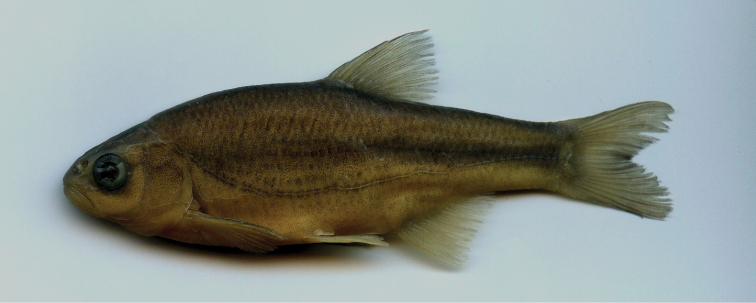
*Alburnoides damghani* sp. n., CMNFI 2015-0091, holotype, female, 67.0 mm SL; Iran, Semnan Prov., Cheshmeh Ali, Damghan River tributary.

*Description of holotype* (Fig. 3). The caudal-fin lobes are rounded and the fin is shallowly forked. A ventral keel between the pelvics and the anal fin is scaleless for 1/3 of the length in front of the anus. There is a pelvic axillary scale and scales extend over the proximal bases of the anal fin forming a sheath. The upper body profile is convex, similar to the lower profile. The body is relatively thick and the caudal peduncle short and deep (its depth enters the length 1.7 times).

The eye is small, its horizontal diameter enters interorbital width 1.2 times. The snout is short and stout, its length only slightly exceeds the eye diameter. The upper jaw slightly projects over the lower jaw. The mouth is small, terminal, the mouth cleft is slightly curved, and the tip of the mouth cleft is on a level with the lower margin of the pupil. The posterior end of the lower jaw is on a vertical with the anterior margin of the pupil. The body depth enters SL 3.2 times, HL enters 3.7, predorsal length 1.8, caudal peduncle depth 7.7, caudal peduncle length 4.7, length of longest dorsal fin ray 4.4, and length of longest anal-fin ray to scale sheath 6.6. Eye horizontal diameter enters HL 3.9 times, snout length enters 3.4, and interorbital width 3.2. Pectoral-fin length enters pectoral-fin origin to pelvic-fin origin distance 1.2 times and pelvic-fin length enters pelvic-fin origin to anal-fin origin distance 1.1 times.

Dorsal-fin rays are 4 unbranched and 8½ branched, anal fin rays are 3 unbranched and 12½ branched, pectoral-fin branched rays are 13, and pelvic-fin branched rays are 7. The anal-fin origin is on a vertical from the posterior end of the dorsal-fin base. Total lateral-line scales number 46 and those to posterior margin of hypurals 44, scales around caudal peduncle 17, scales above lateral line to dorsal fin origin are 9, scales below lateral line to anal-fin origin are 4, scales below lateral line to pelvic-fin origin are 4, and midline predorsal scales are 27. Pharyngeal teeth 2.5-4.2. Gill rakers number 6, they are short and stubby, the longest touching the adjacent one when appressed. Total vertebrae number 40 (abdominal vertebrae 20, caudal vertebrae 20). Predorsal vertebrae number 13.

The peritoneum is silvery with fine melanophores. The lateral line is clearly delineated by darker pigment above and below, but this is obscured on the caudal peduncle by the flank stripe. Some pigment on flank scales above the lateral line give the impression of stripes. A mid-flank stripe is evident, darkest on the caudal peduncle. The back and top of the head are dark, the belly is light with almost no melanophores. Melanophores are dense dorsally on the flank becoming progressively less ventrally. All fins have melanophores lining the rays, and the dorsal, anal and caudal fins have melanophores on the membranes, with very few melanophores on the pectoral- and pelvic-fin membranes. The unbranched pectoral-fin ray is lined with melanophores on its inner margin.

**Figure 4.**
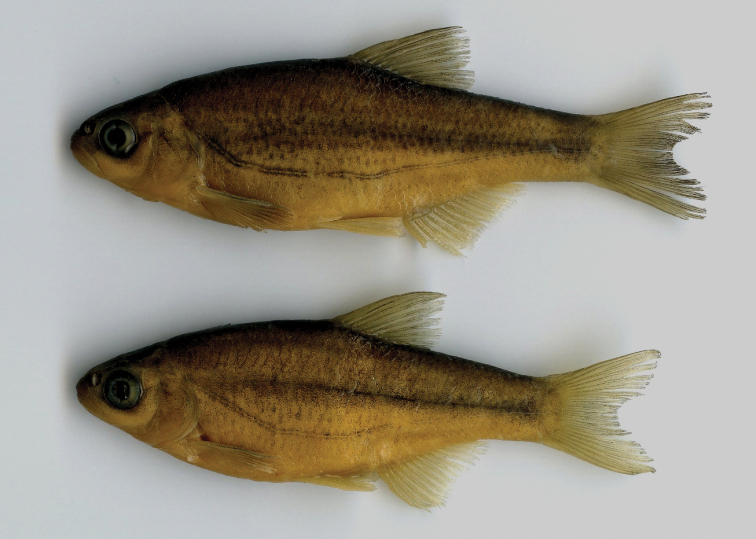
*Alburnoides damghani* sp. n., paratypes CMNFI 2015-0091A, a, 67.6 mm SL, b, 60.5 mm SL, Iran, Semnan Prov., Cheshmeh Ali, Damghan River tributary.

**Figure 5.**
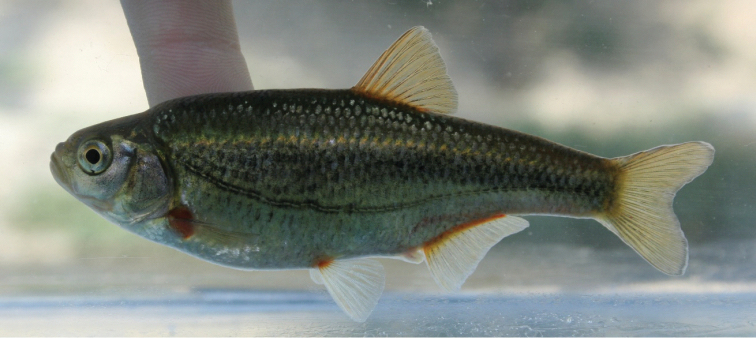
Live specimen of *Alburnoides damghani* sp. n., Iran, Semnan Prov., Cheshmeh Ali, Damghan River tributary.

*Description of paratypes.* General appearance of body is shown in Figures 2–4 and morphometric data are given in Table 3. Body compressed but thick, upper body profile clearly convex, similar to the lower profile. The eye is small, always less than interorbital width (eye horizontal diameter enters interorbital width 1.1−1.4 times). Snout short and stout, only slightly pointed, snout length about equal to eye horizontal diameter. Mouth short, posterior end of upper jaw commonly in front of vertical with anterior margin of eye, posterior end of lower jaw on about vertical with anterior margin of pupil. Mouth terminal, but mouth cleft more or less markedly curved and tip of mouth cleft is on or below a level from lower margin of the pupil. Upper jaw slightly produced over lower jaw in most specimens, especially larger-sized. Ventral keel between pelvic and anal fin not sharp and weakly pronounced, variably scaled (examined in 24 paratypes): completely scaleless (in 7 specimens), scaleless along 3/4 (4 specimens), 2/3 (4 specimens), 1/2 (5 specimens), 1/4 (2 specimens), 1/5 (1 specimen) of keel length in front of the anus or completely scaled (1 specimen). Pelvic axillary scale present extending over the proximal base of the anal fin. Caudal fin shallowly forked with rounded lobes. Anal-fin origin at the vertical of the posterior end of the dorsal fin base (Fig. 5) or in front of it (Fig. 4). The dorsal-fin outer margin is truncate to slightly convex and the anal-fin outer margin is slightly concave. For measurement and ratios see Table 4.

**Table 4.** Morphometric data for the holotype of *Alburnoides damghani* (CMNFI 2015-0091) and paratypes (CMNFI 2015-0091A, n=24). Holotype data is included in the range and mean values.

Character	Holotype	Min	Max	Mean	SD
SL, mm	67.0	54.6	84.4		
Body depth at dorsal-fin origin (% SL)	30.9	28.9	33.3	31.14	1.16
Depth of caudal peduncle (% SL)	12.9	12.0	14.1	13.01	0.51
Depth of caudal peduncle (% length of caudal peduncle)	60.6	57.3	68.1	63.10	2.91
Body width at dorsal-fin origin (% SL)	12.5	12.3	15.9	14.32	1.01
Caudal peduncle width (% SL)	4.6	3.9	5.6	4.66	0.42
Predorsal length (% SL)	54.9	53.0	57.1	55.12	1.23
Postdorsal length (% SL)	35.4	33.2	40.2	35.34	1.64
Prepelvic length (% SL)	49.1	45.9	53.2	49.15	1.44
Preanal length (% SL)	66.3	62.9	69.7	66.38	1.52
Pectoral – pelvic-fin origin length (% SL)	23.6	21.4	27.2	23.85	1.49
Pelvic – anal-fin origin length (% SL)	19.6	16.6	20.6	18.37	1.11
Length of caudal peduncle (% SL)	21.4	19.0	22.4	20.64	0.85
Dorsal-fin base length (% SL)	14.0	11.6	19.7	13.71	1.57
Dorsal fin depth (% SL)	22.5	18.3	23.9	20.93	1.29
Anal-fin base length (% SL)	17.1	14.7	19.5	17.45	1.42
Anal fin depth (% SL)	15.0	12.3	15.2	13.74	0.90
Pectoral-fin length (% SL)	19.9	17.7	21.5	19.66	1.00
Pelvic-fin length (% SL)	17.1	13.3	18.7	16.39	1.17
Head length (% SL)	27.2	24.5	28.1	26.74	0.88
Head length (% body depth)	87.9	77.6	92.4	85.96	3.54
Head depth at nape (% SL)	21.1	19.0	22.5	21.07	0.96
Head depth at nape (% HL)	77.8	73.6	83.7	78.83	2.79
Head depth through eye (% HL)	54.9	52.5	66.4	57.71	3.16
Maximum head width (% SL)	13.3	12.2	14.9	13.68	0.61
Maximum head width (% HL)	49.0	48.4	56.5	51.21	2.27
Snout length (% SL)	7.8	6.5	7.9	7.33	0.35
Snout length (% HL)	28.9	24.4	29.3	27.42	1.11
Eye horizontal diameter (% SL)	6.9	6.5	7.9	7.04	0.38
Eye horizontal diameter (% HL)	25.5	23.5	28.2	26.35	1.36
Eye horizontal diameter (% interorbital width)	81.6	71.3	87.8	78.22	4.40
Postorbital distance (% HL)	47.8	47.8	53.6	50.81	1.68
Interorbital width (% SL)	8.5	7.8	9.7	9.02	0.49
Interorbital width (% HL)	31.3	31.3	36.2	33.72	1.44
Length of upper jaw (% HL)	28.1	28.1	35.3	31.81	1.65
Length of upper jaw (% SL)	7.6	7.5	9.8	8.51	0.54
Length of lower jaw (% SL)	11.2	9.7	12.4	10.99	0.64
Length of lower jaw (% HL)	41.2	37.4	44.6	41.10	1.70
Length of lower jaw (% interorbital width)	131.6	109.8	142.8	122.09	7.29
Length of lower jaw (% depth of operculum)	94.3	90.7	104.3	96.87	4.31
Depth of operculum (% HL)	43.7	38.5	46.3	42.47	1.82
**Ratios**					
Interorbital width/eye horizontal diameter	1.2	1.1	1.4	1.28	0.07
Snout length/eye horizontal diameter	1.1	1.0	1.1	1.04	0.05
Head depth at nape/eye horizontal diameter	3.0	2.8	3.2	3.00	0.13
Head length/caudal peduncle depth	2.1	1.9	2.3	2.06	0.08
Length of caudal peduncle/caudal peduncle depth	1.6	1.5	1.7	1.59	0.07
Length of lower jaw/caudal peduncle depth	0.9	0.8	1.0	0.85	0.05
Pectoral-fin length/pectoral – pelvic-fin origin distance	0.8	0.7	1.0	0.83	0.08
Predorsal length/head length	2.0	1.9	2.2	2.06	0.07

In 24 paratypes (CMNFI 2015-0091): the lateral line is complete with 1 or 2 unpored scales at the posterior end of the lateral series, total lateral-line scales 40 (1), 41 (1), 42 (4), 43 (3), 44 (7), 45 (3), 46 (2) or 47 (1), lateral-line scales to the margin of hypurals 40 (2), 41 (3), 42 (7), 43 (5), 44 (1), 45 (3) or 46 (1), total gill rakers in the outer row on first left arch number 6 (5), 7 (16) or 8 (3), gill rakers are rather thick, short and widely spaced, not touching the adjacent raker base when appressed, pharyngeal tooth counts are 2.5-4.2 in 19 specimens from 25 examined and 2.5-4.1 in 5 specimens. The general topography of cephalic sensory canals and numbers of pores is typical of most *Alburnoides* (e.g., Coad and Bogutskaya 2009). The supraorbital canal is not lengthened in its posterior section and has 8-10, commonly 9 pores with 2−4, commonly 3, and 5−7, commonly 6, canal openings on the nasal and frontal bones, respectively. The infraorbital canal has 13−17, commonly 14−15, pores with 4 (rarely 3 or 5) canal openings on the first infraorbital. The preopercular-mandibular canal is complete, with 13-17, modally 14-16, pores and commonly 5 or 6 and 8 or 9 canal openings on the dentary and preoperculum, respectively. The supratemporal canal is complete, with 5 (rarely 6 or 7) pores.

**Figure 6.**
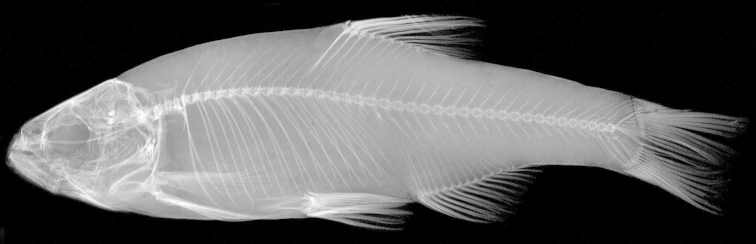
*Alburnoides damghani* sp. n., radiograph of a paratype (ZM-CBSU 2011-1) showing 20+20 vertebral formula.

In 39 paratypes (CMNFI 2015-0091 and ZM-CBSU 2011-1): dorsal-fin unbranched rays 3 or 4 (in 4 specimens only), branched dorsal-fin rays 7½ (5), 8½ (33) or 9½ (1) (mean 7.9 [without ½], sd 0.5). Anal-fin unbranched rays 3, branched anal-fin rays 10½ (2), 11½ (11), 12½ (20) or 13½ (6) (11.8 [without ½], sd 0.8). Total vertebrae number 39 (4), 40 (28) or 41 (7) (40.1, 0.5). Predorsal vertebrae number 12 (5), 13 (26) or 14 (8) (13.1, 0.6). Abdominal vertebrae number 20 (31) or 21 (8) (20.2, 0.4). Caudal vertebrae number 19 (8), 20 (28) or 21 (4) (19.9, 0.5). The vertebral formulae are 20+20 (in 24 specimens, Fig. 6), 21+19 (5), 20+21 (4), 20+19 (3), 21+20 (3), 20+19 (1) or 19+20 (1). Thus, the caudal vertebral region most commonly is equal to the abdominal region (in 23 paratypes) or longer than the latter (in 11), the difference between abdominal and caudal counts being +2 (5), +1 (6), 0 (23) or –1 (5).

Mature males bear tubercles on the unbranched and branched fin rays, in a single row branching into two distally on the branched rays. These are most prominent on the pectoral, pelvic and anal fins. Tubercles line scale margins in a single row of up to six tubercles, in particular over the anal fin and on the lower caudal peduncle. Scales below the dorsal fin are also lined with tubercles but to a much lesser extent than those above the anal fin. Flank scales generally may bear tubercles but many do not and anterior flank scales may have only a single tubercle. Minute tubercles are present on the dorsal and upper head surface.

#### Coloration of live specimen.

Pigmentation consists of a darker back fading to a silvery white belly, three to four rows of large dark spots above lateral line starting from posterior part of operculum to posterior level of anal fin, continuing with two rows behind anal fin to base of caudal fin, small black spots on the operculum, behind and below the eye, smaller and less dark spots between the eye and upper jaw, a lateral line demarcated by pigment above and below it (the typical “stitched” pattern in many *Alburnoides* species), base of anal, pelvic, pectoral and dorsal fins almost reddish-orange, caudal-fin base pale or faint yellow. Posterior free margin of dorsal, anal, caudal and pelvic fins whitish hyaline, faint pigmentation on the caudal-fin centre branching distally to follow the inner margins of the fin fork, and fine pigmentation on the proximal part of dorsal- and anal-fin rays, darker in dorsal-fin rays (Figs 3, 4).

#### Etymology.

The species name links to the type locality, Damghan (Cheshmeh Ali, Damghan River tributary). Proposed common name: Damghan riffle minnow, Mahi-e-Khayateh-e-Damghan (Farsi).

#### Distribution and conservation.

*Alburnoides damghani* sp. n. has only been collected from its type locality, Cheshmeh Ali in the Damghan River system, north Dasht-e Kavir Basin (N-Iran) (Fig. 1). *Aphanius kavirensis* Esmaeili, Teimori, Gholami & Reichenbacher, 2014 which is restricted to this spring, co-exists with *Alburnoides damghani* sp. n. (Esmaeili et al. 2010, 2014a,b). Its restricted range, drought in recent years and introduction of the exotic carnivorous fish, *Oncorhynchus mykiss* (Walbaum, 1792) (personal observation of HRE) may threaten this endemic species.

#### Habitat

**Figure 7.**
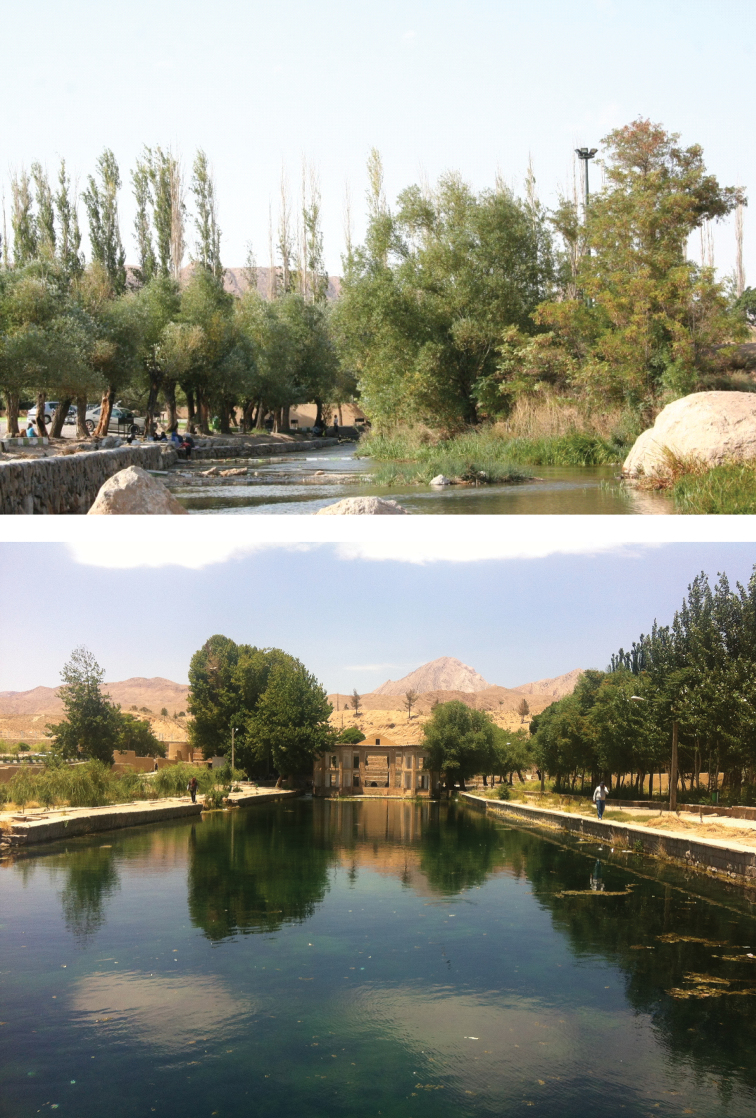
Two views of Cheshmeh Ali, Damghan, type locality of *Alburnoides damghani* sp. n.

**(Fig. 7).** At the Cheshmeh Ali sampling site, the spring was about 5−10 m wide, with substrate consisting of coarse gravel and boulders, good riparian vegetation and almost fast-flowing and transparent waters. The physicochemical parameters at the spot were: dissolved oxygen, 7.54 mg/L, total dissolved solids, 318 mg/L, salinity, 0.32‰, conductivity, 552 μm/cm, pH: 7.97 and water temperature 23.25 °C.

#### Comparative remarks.

*Alburnoides damghani* sp. n., together with other Iranian species of the genus, belongs to the south-eastern group of species distributed in the eastern area of the genus distribution and characterised by commonly 4 pharyngeal teeth in the long row on the right 5th ceratobranchial (Bogutskaya and Coad 2009). As most distinguishing characters for species identification are counts (numbers of branched rays in the dorsal and anal fins, gill rakers, lateral-line scales and vertebral counts), a MDS statistical analysis was performed based on mean values of these counts (Table 5) to visualize the level of similarity of individual samples (species) in the Caspian Sea basin localities and adjacent endorheic basins. Frequences of occurrence of individual counts by characters can be found in earlier publications (Bogutskaya and Coad 2009, Coad and Bogutskaya 2009, 2012, Mousavi-Sabet et al. 2015a, b). The map plotting each sample in two-dimensional space is presented in Fig. 9; stress value is 0.04 (very low) meaning that the results are highly reliable (Davison 1983). The proximity of the examined samples to each other indicate how similar they are, and *Alburnoides damghani* sp. n. stands far apart from all other species, being relatively closer to *Alburnoides namaki*, *Alburnoides varentsovi* and *Alburnoides* sp. (Amu Darya River), morphologically.

**Figure 8.**
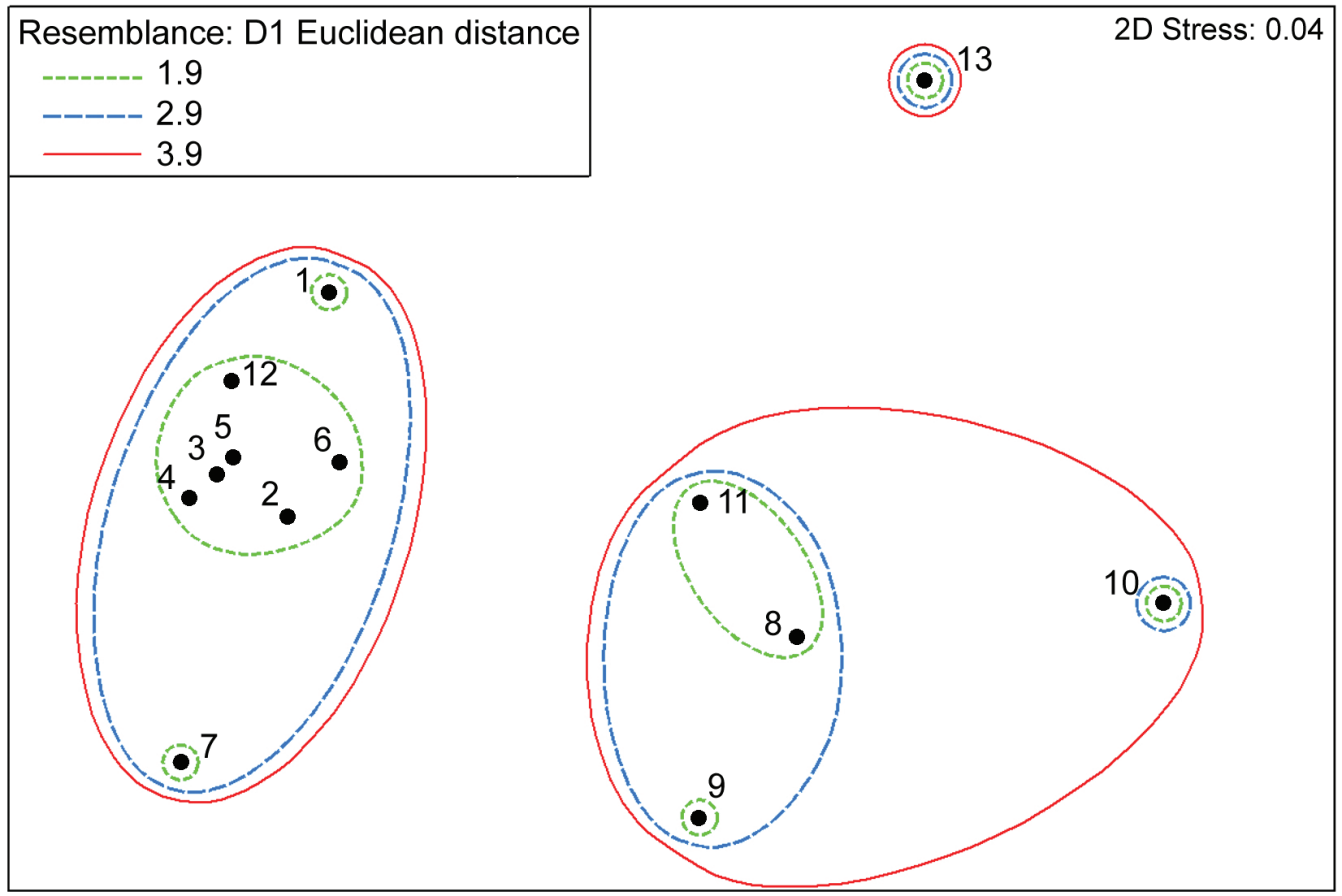
Results of a DMS analysis showing observed similarities/dissimilarities (distances) between the examined groups of samples of *Alburnoides*, from the Caspian Sea basin and adjacent endorheic basins, based on meristic characters (Table 5). **1** *Alburnoides eichwaldii* **2** Alburnoides cf. eichwaldii: west of Safid River **3** *Alburnoides samiii* **4** *Alburnoides tabarestanensis* **5** *Alburnoides parhami* **6** *Alburnoides parhami* (data from Mousavi-Sabet et al. 2015b) **7** *Alburnoides holciki* **8** *Alburnoides varentsovi* **9** *Alburnoides* sp. Amu Darya River **10** *Alburnoides damghani* sp. n. **11** *Alburnoides namaki* **12** *Alburnoides coadi* (data from Mousavi-Sabet et al. 2015b) **13** *Alburnoides petrubanarescui*.

**Figure 9.**
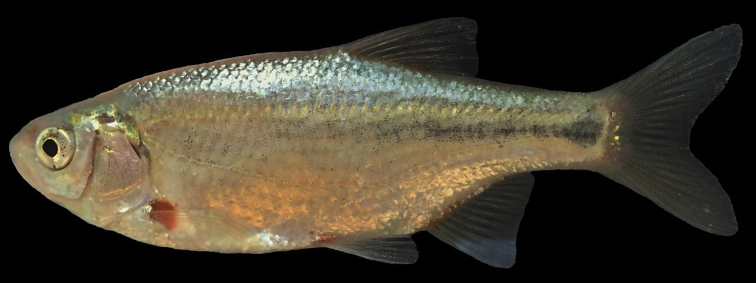
Uncatalogued *Alburnoides coadi*, 84.0 mm SL; Iran, Tehran Prov., Nam River, at Firoz Koh, 35°43'20.8"N 52°39'20.0"E.

**Table 5.** Mean values of some meristic characters of *Alburnoides* species from the Caspian Sea basin and adjacent endorheic basins, used for the DMS analysis. Numbers of samples as in Figs 1 and 8 (own data except for two indicated samples).

	Species	Branched anal-fin rays (without «½»)	Branched dorsal-fin rays (without «½»)	Gill rakers	Lateral-line scales to margin of hypurals	Total vertebrae	Predorsal vertebrae	Abdominal vertebrae	Caudal vertebrae	Difference between abdominal and caudal vertebral counts
**1**	*Alburnoides eichwaldii* (n=160)	12.16	7.95	7.37	48.87	41.25	13.65	20.72	20.53	0.19
**2**	Alburnoides cf. eichwaldii: west of Safid River (n=44)	13.16	8.00	7.95	48.50	40.57	13.18	20.13	20.41	-0.25
**3**	*Alburnoides samiii* (n=113)	12.87	8.00	8.62	48.96	40.26	12.63	19.89	20.37	-0.48
**4**	*Alburnoides tabarestanensis* (n=21)	12.82	7.95	8.58	49.02	40.27	12.18	19.73	20.55	-0.82
**5**	*Alburnoides parhami* (n=35)	13.11	7.86	8.14	49.12	40.29	12.66	20.09	20.26	-0.14
**6**	*Alburnoides parhami* (n=50; from Mousavi-Sabet et al. 2015b)	12.38	7.89	7.62	48.64	40.24	12.30	20.08	20.22	-0.14
**7**	*Alburnoides holciki* (n=18)	14.72	8.23	7.22	48.81	41.05	13.21	19.89	21.16	-1.21
**8**	*Alburnoides varentsovi* (n=55)	12.53	7.90	6.70	45.10	39.93	12.24	19.78	20.15	-0.36
**9**	*Alburnoides* sp. Amu Darya River (n=30)	13.43	8.00	6.50	45.40	40.90	12.93	19.77	21.17	-1.40
**10**	*Alburnoides damghani* (n=40)	11.77	7.88	6.88	42.65	40.08	13.08	20.18	19.90	0.28
**11**	*Alburnoides namaki* (n=48)	11.83	8.11	7.00	46.12	39.72	12.22	19.80	19.90	-0.10
**12**	*Alburnoides coadi* (n=50; from Mousavi-Sabet et al. 2015b)	12.38	7.92	8.54	48.88	39.88	13.26	19.76	19.94	-0.18
**13**	*Alburnoides petrubanarescui* (n=30)	9.30	7.30	7.22	45.62	40.53	13.44	21.00	19.54	1.44

When compared to *Alburnoides* species distributed in the Caspian Sea basin and adjacent endorheic basins in Iran, *Alburnoides damghani* sp. n. is clearly different from *Alburnoides parhami* from the Atrek River drainage by having four teeth in the long row on the 5^th^ ceratobranchial (vs. 5). By having five pharyngeal teeth in the long row on the 5^th^ ceratobranchial (this character state is invariably present in all examined specimens), *Alburnoides parhami* stands apart from all other species in Iran. Besides the number of teeth, *Alburnoides damghani* sp. n. is distinguished from *Alburnoides parhami* by having three unbranched dorsal fin rays (vs. often four, found in 13 from 35 examined specimens), commonly a partly scaleless ventral keel (vs. sharp and commonly scaleless), a terminal mouth with the tip of the mouth cleft on or below a level from lower margin of the pupil (vs. an upturned terminal mouth with the tip of the mouth cleft on a level with the upper half of the pupil), and 40−46 lateral-line scales to the margin of the hypurals (vs. 45−51).

*Alburnoides damghani* sp. n. differs from both *Alburnoides petrubanarescui* (which is the most morphologically peculiar species in the area possessing the lowest number of anal-fin branched rays) and *Alburnoides namaki* (a species phylogenetically close to *Alburnoides damghani*, see Fig. 2) by a slightly pointed snout (vs. markedly rounded), a terminal mouth with the tip of the mouth cleft on or below a level from lower margin of the pupil (vs. subterminal, with the tip of the mouth cleft on or below a level from lower margin of the eye), and 40−46 lateral-line scales (to the margin of the hypurals) (vs. 42−51, commonly 44−48). *Alburnoides damghani* sp. n. further differs from *Alburnoides petrubanarescui* by commonly 8½ branched dorsal-fin rays (vs. commonly 7½), commonly 11−12½ branched anal-fin rays (vs. commonly 9½), abdominal vertebrae commonly 20 (vs. commonly 21), and a ventral keel commonly partly or completely scaleless (vs. completely scaled). From *Alburnoides namaki*, *Alburnoides damghani* sp. n. can be further distinguished by a smooth and sometimes partly scaled ventral keel (vs. sharp and completely scaleless) and a higher number of predorsal vertebrae (modally 13 vs. modally 12).

*Alburnoides coadi* (Fig. 9) is the phylogenetically closest sister to *Alburnoides namaki* and the two species are rather similar in shape of the head, mouth and body; however, the two species are different by a complex of meristic characters (Fig. 9). *Alburnoides damghani* sp. n. differs from *Alburnoides coadi*, first of all, by a lower number of the lateral-line scales to the margin of the hypurals (40−46 vs. 47−51), a higher number of gill rakers (8−10, modally 8 and 9 vs. 6−8, modally 7), and a lower number of total vertebrae (modally 40 vs. modally 41).

*Alburnoides damghani* differs from *Alburnoides holciki* and *Alburnoides qanati* by a relatively small-sized eye with horizontal diameter slightly to markedly less than interorbital width (vs. large eye with eye diameter about equal to or larger than interorbital width), a tip of the mouth cleft on a level with or below the lower margin of the pupil (vs. on a level with the upper half to the upper margin of the pupil), and a shallowly forked caudal fin with rounded lobes (vs. clearly forked caudal fin with pointed lobes). *Alburnoides damghani* sp. n. is further distinguished from *Alburnoides holciki* from the Hari River drainage in northeastern Iran by a usually smooth and often partly scaled ventral keel (vs. sharp and scaleless), a lower number of total lateral-line scales (44−47 vs. 47–57), a lower number of anal-fin rays (commonly 11−12½ vs. 13–16½), a lower number of total vertebrae (39−41, usually 40 vs. 40–42, usually 41), an abdominal vertebral region most commonly equal to or longer than caudal region, and most common vertebral formulae 20+20 and 21+19 (vs. abdominal region shorter than caudal region, and most common vertebral formulae 20+21, 20+22 and 19+21). *Alburnoides damghani* further differs from *Alburnoides qanati* (the Pulvar River drainage of Fars Province in southern Iran) by modally 12½ branched dorsal-fin rays (vs. modally 11½).

The new species differs from *Alburnoides eichwaldii* by a lower number of total lateral-line scales, 44−47 (vs. 44−56, commonly over 47), a lower number of gill rakers, 6−8 (vs. 6−10. commonly 8 and 9), a lower number of total vertebrae, 39−41 with a mode of 40 (vs. (38, 39)40-43 with a modal range of 41−42), a lower number of adbominal vertebrae with a clear mode of 20 (vs. clear mode of 21), a lower number of predorsal vertebrae, 12−14 with a mode of 13 (vs. 13−15 with a mode of 14), and the most common vertebral formulae 20+20 and 21+19 (vs. 21+21, 21+20 and 20+21).

*Alburnoides damghani* sp. n. can be distinguished from *Alburnoides tabarestanensis* from the type locality (the Tajan River) by a commonly partly scaled keel (vs. a commonly completely scaleless ventral keel), a lower number of total lateral-line scales (44−47 vs. 47−52), commonly 11−12½ branched anal-fin rays (vs. 12−14½, with a mode of 13½, branched anal-fin rays), and a greater head depth at nape (74−84% HL vs. 73−75% HL).

As can be seen from Fig. 8, *Alburnoides* sp. from rivers in the south of the Talysh Mountains and west of the Safid River (examined samples are mostly from estuarine areas of the rivers in Gilan Province), *Alburnoides samiii* from the type locality (Safid River drainage), and *Alburnoides tabarestanensis* from different localities (other than the type one) cannot be clearly discriminated by their meristic character states. Also, they are rather similar by the head and body shape, having most commonly a horizontal, slightly curved mouth, with a tip of the mouth cleft often on a level below the lower margin of the pupil, a slightly to markedly rounded snout, a variably but commonly well forked caudal fin. The ventral keel in these species is partly to completely scaled, considerably varying in and between samples. Discussion on morphological differences between these species/groups of populations is beyond the scope of this paper; *Alburnoides damghani* sp. n. clearly differs from this complex by having a lower number of lateral-line scales, 40−46 to posterior margin of hypural (vs. 42−56, commonly over 45, averaging 48−49).

#### Comparative material.

Extensive comparative material is listed in Bogutskaya and Coad (2009) and Coad and Bogutskaya (2009, 2012). Data for *Alburnoides coadi* (Nam River) and *Alburnoides parhami* (Baba-Aman stream) from the type localities are taken from Mousavi-Sabet et al. (2015b). Additional material: *Alburnoides eichwaldii* ZM-CBSU 2007(1386a), 20, Iran, Ardabil Prov., Almas River, Aras River system, Caspian Sea basin, 38°09'31"N, 48°11'37"E, 3 October 2007, coll. H.R. Esmaeili; *Alburnoides samiii* ZM-CBSU 2009 (1388a), 29, Iran, Gilan Prov., Safid Rud River, at Emamzade Hashem, 37°01'11"N, 49°38'E, 29 June 2009, coll. H.R. Esmaeili, S. Babai; *Alburnoides samiii* ZM-CBSU A189-210, 21, Iran, Mazandaran Prov., Siah River at Sarookolah, Caspian Sea basin, 36°27'13"N, 52°53'38"E, 29 June 2009, coll. H.R. Esmaeili; Alburnoides cf. tabarestanensis ZM-CBSU 2009(1388b), 15, Iran, Mazandaran Prov., Keslian River, Talar River drainage, at Shirgah, Caspian Sea basin, 36°18'15"N, 52°53'07"E, 31 June 2009, coll. H.R. Esmaeili, H. Mostafavi, Alburnoides Teimori, Alburnoides Gholamifard; Alburnoides cf. tabarestanensis ZM-CBSU 2011(1389), 25, Iran, Mazandaran Prov., Shirin River, Caspian Sea basin, 36°08'59"N, 53°50'02"E, 9 November 2011, coll. H. Mostafavi; Alburnoides cf. tabarestanensis ZM-CBSU 2007(1386b), 10, Iran, Golestan Prov., Gorgan River at Zaringol, Caspian Sea basin, 36°50'39"N, 54°58'24"E, 6 August 2007, coll. H.R. Esmaeili; *Alburnoides parhami* CMNFI 2016-0050, 25 , Iran, Khorasan-e Shomali Prov., Tabarak Dam, Atrak River tributary, Ghoochan, Caspian Sea basin, 37°08'09"N, 58°40'44"E, 25 August 2011 coll. H.R. Esmaeili, Alburnoides Gholamifard, G. Sayyadzadeh, R. Zamaniannejad.

## Discussion

The present data comprise the first comprehensive molecular study based on the COI barcode region on the genus *Alburnoides* in Iran and will serve as a reference for future studies of this diverse taxon. Based on the reconstructed phylogenetic trees, 4 major lineages were formed, which is well supported by high posterior probability and bootstrap values in seven groups (Fig. 2): I) *Alburnoides strymonicus* − *Alburnoides thessalicus* lineage, II) *Alburnoides bipunctatus* − *Alburnoides ohridanus* − *Alburnoides prespensis* group lineage, III) *Alburnoides* sp. lineage (Greece: Sperchios drainage) and IV) Iranian *Alburnoides* lineage (*Alburnoides eichwaldii* lineage).

Lineage I includes two species, *Alburnoides strymonicus* (originally described from the Toplitza River and the Struma River, Bulgaria) and *Alburnoides thessalicus* (rivers Spinios and Sperchios, Greece). Based on the phylogenetic tree represented here, both of them are distinct monophyletic (posterior probability of 1 or 100) species in the genus *Alburnoides*.

Lineage II comprises highly diverse *Alburnoides* species including *Alburnoides bipunctatus*, *Alburnoides ohridanus* and three close related species, *Alburnoides devolli*, *Alburnoides fangfangae* and *Alburnoides prespensis*. *Alburnoides bipunctatus* was originally described from the Weser River near Minden, Germany. Based on our COI data, it is sister to *Alburnoides ohridanus* plus a group of three closely related species, *Alburnoides devolli*, *Alburnoides fangfangae*, and *Alburnoides prespensis*. *Alburnus bipunctatus ohridanus* from Lake Ohrid was elevated to the species level based on morphological characters by Kottelat and Freyhof (2007), Coad and Bogutskaya (2009), Bogutskaya and Coad (2009), Bogutskaya et al. (2010), and Turan et al. (2014). This is supported here based on COI sequences available in NCBI (from the Erzen River and Ohrid Lake in Albania). In the *Alburnoides prespensis* species group of Lineage II, there are three related species: *Alburnoides prespensis*, *Alburnoides devolli* and *Alburnoides fangfangae*. *Alburnus bipunctatus prespensis* was described from Lake Prespa and its tributaries, Republic of Macedonia and it was morphologically considered as a valid species by Kottelat and Freyhof (2007), Coad and Bogutskaya (2009), Bogutskaya and Coad (2009), Bogutskaya et al. (2010) and Turan et al. (2014). It is genetically supported by Perea et al. (2010) and here, based on COI gene sequences available in NCBI (all from Lake Prespa drainages). *Alburnoides devolli* was described based on morphological and meristic characteristics from the upper Devoll River system, Albania (Adriatic Sea basin) (Bogutskaya et al. 2010). Based on the reconstructed phylogenetic tree (Fig. 1) using the available COI data, it seems that 4 COI sequences of collected specimens from the Devoll drainage nests within *Alburnoides prespensis* and *Alburnoides fangfangae* and thus its validity is not supported by the COI barcode region. *Alburnoides fangfangae* was described from the upper Osum River system, Albania (Adriatic Sea basin) based on morphological and meristic characteristics (see Bogutskaya et al. 2010). However, its available sequences from the Osumi drainage, Albania are nested within the *Alburnoides prespensis* and *Alburnoides devolli* group (see also Stierandová et al. 2016) and thus its validity is not supported by the COI barcode region.

Lineage III comprises one monotypic undescribed species (accession number: KJ552427, Greece: Sperchios drainage).

Lineage IV is formed by the highly diverse *Alburnoides* species from the southern Caspian Sea, Tigris River, Namak Lake, Dasht-e Kavir, Kor River and Hari (= Tedzhen) River basins and it is comprised of a monophyletic group with high posterior probability of 1. This lineage might be called the *Alburnoides eichwaldii* species group as some of them had been considered as *Alburnoides bipunctatus eichwaldii*. In this lineage, *Alburnoides holciki* is a sister (supported with a high posterior probability of 1) to all other species including *Alburnoides eichwaldii* plus *Alburnoides qanati* (the most northern and southern *Alburnoides* species of Iran respectively) and a group comprising *Alburnoides idignensis*, *Alburnoides nicolausiAlburnoides tabarestanensis*, *Alburnoides samiii*, *Alburnoides damghani* sp. n., and *Alburnoides namaki* (Fig. 2). Two species from the Tigris River basin, *Alburnoides idignensis* and *Alburnoides nicolausi*, are very closely related and are not well supported as sister taxa (low posterior probability of 0.62). However, the ancestral node for *Alburnoides idignensis* is 1.0, as is the ancestral node for *Alburnoides nicolausi*, which is strong support for monophyly of each of these species.

Lineage IV, *Alburnoides damghani* sp. n. (Damghan River drainage, Dasht-e Kavir basin) is sister (posterior probability = 0.999) to *Alburnoides coadi* from the Nam River, a tributary of the Hableh River drainage, Dasht-e Kavir basin) (Fig. 9) and *Alburnoides namaki* from the Qareh Chai River drainage (Namak Lake basin). It has already been reported that Hableh River (Dasht-e Kavir basin) fish elements are much closer to those from the Qom River drainage (Namak Lake basin) than to the other river systems of the Dasht-e Kavir basin (Freyhof et al. 2014) which is supported here. The validity of *Alburnoides eichwaldii* from the Kura River is supported by the COI barcode region. *Alburnoides bipunctatus armeniensis* Dadikyan, 1972 from Rivers Arpa, Vorotan, Vedi, Marmarik, Kasakh, and their tributaries (Aras River system, Kura River drainage) is a synonym of *Alburnoides eichwaldii* according to Bogutskaya and Coad (2009) being supported here by using COI barcode region of four fresh collected specimens from two localities in the Aras River (near the cities of Poldasht and Parsabad, border of Iran and Azerbaijan (Fig. 1). Recently, the phylogenetic relationships and taxonomy in the genus *Alburnoides* have been examined by comparative sequencing analyses of mitochondrial and nuclear markers by Stierandová et al. (2016). According to these authors, a molecular analysis revealed 17 Eurasian lineages divided into two main clades, termed the Ponto-Caspian and European in accordance with the lineage distribution. According to Stierandová et al. (2016) the European clade is represented by *Alburnoides bipunctatus*, *Alburnoides rossicus*, *Alburnoides tzanevi, Alburnoides maculatus, Alburnoides ohridanus, Alburnoides strymonicus*, 4 unnamed or undescribed species and populations defined as the *Alburnoides prespensis* complex including *Alburnoides prespensis* s. stricto, *Alburnoides fangfangae* and *Alburnoides devolli*. However, they concluded that phylogenetic analyses present ambiguous results and do not support recently accepted taxonomy which presumes validity of three species: *Alburnoides prespensis, Alburnoides fangfangae*, and *Alburnoides devolli* supporting our results, considering *Alburnoides fangfangae* and *Alburnoides devolli* being part of an *Alburnoides prespensis* complex (Fig. 2). Furthermore, Stierandová et al. (2016) considered *Alburnoides eichwaldii*, *Alburnoides fasciatus*, *Alburnoides kubanicus*, Safid River population (now *Alburnoides samiii*) and Talar population (now *Alburnoides tabarestanensis*) in the Ponto-Caspian clade. Base on the current study, IV lineage can be considered in the Ponto-Caspian clade and I and II lineages both in the European clade. Moreover, the placements of *Alburnoides strymonicus* and *Alburnoides* sp. Sperchios, which were uncertain in Stierandová et al. (2016) appear to be well-supported here. From a biogeographical viewpoint, the locations of lineage richness in most cases correspond to confirmed glacial refugia (Stierandová et al. 2016).

To conclude, the genetic analyses supported the validity of many morphologically distinguishable species of the genus *Alburnoides* in Iran (i.e., *Alburnoides damghani* sp. n., *Alburnoides eichwaldii, Alburnoides holciki, Alburnoides namaki, Alburnoides qanati*) belonging to a distinct phylogenetic lineage. Two species of Tigris river basin, *Alburnoides idignensis* and *Alburnoides nicolausi* are very closely related and are not well supported as sister taxa (low posterior probability of 0.62) by the COI barcode region, however, the ancestral node for *Alburnoides idignensis* is 1.0, as is the ancestral node for *Alburnoides nicolausi*, which is strong support for monophyly of each of these species. The analysis also demonstrated the existence of four major phylogenetic lineages within the genus *Alburnoides* in general.

## Supplementary Material

XML Treatment for Alburnoides damghani
